# Environmental-Friendly Modifications of Zeolite to Increase Its Sorption and Anion Exchange Properties, Physicochemical Studies of the Modified Materials

**DOI:** 10.3390/ma12193213

**Published:** 2019-09-30

**Authors:** Jolanta Cieśla, Wojciech Franus, Małgorzata Franus, Karolina Kedziora, Justyna Gluszczyk, Justyna Szerement, Grzegorz Jozefaciuk

**Affiliations:** 1 Institute of Agrophysics, Polish Academy of Sciences, Doswiadczalna 4, 20-290 Lublin, Poland; j.ciesla@ipan.lublin.pl (J.C.); karolinaa.kedzioraa@gmail.com (K.K.); justynagluszczyk@wp.pl (J.G.); j.szerement@ipan.lublin.pl (J.S.); jozefaci@ipan.lublin.pl (G.J.); 2 Department of Geotechnical Engineering, Faculty of Civil Engineering, Lublin University of Technology, Nadbystrzycka 40, 20-618 Lublin, Poland; m.franus@pollub.pl

**Keywords:** clinoptilolite, soil, water vapor adsorption, specific surface area, porosity, isoelectric point

## Abstract

Zeolites, naturally possessing a high negative surface charge and large specific surface, are used in agriculture as cationic fertilizers, water holders, heavy metals, and organic pollutants sorbents. Since some nutrients occur in anionic forms, there is a need to modify the zeolite surface to hold anions. In this study, hydrogen (hydrochloric acid), iron (Fe^2+^ and Fe^3+^), and aluminum cations as well as the influence of sodium hydroxide modifiers on the specific surface area, water vapor, adsorption energy, fractal dimension, mesopore volumes and radii, electrokinetic (zeta) potential, and isoelectric point were investigated. The use of alkali solution did not affect the zeolite properties significantly, whereas hydrogen, iron, and treatments with aluminum cations resulted in an increase in the specific surface area, mesopore volumes, and radii, and a decrease in the water-binding forces. Aluminum cations were the most effective in recharging the zeolite surface from negative to positive, shifting the isoelectric point toward the highest values. Calcination enlarged the negative surface charge and mesopore radius, and diminished the surface area and mesopore volume. The modified zeolites are promising carriers of anionic nutrients, large surface area sorbents, and suppliers of water for plant roots in soil.

## 1. Introduction

Alongside their widespread applications in the chemical industry, microelectronics, optics, medicine livestock nutrition, and many other areas [1,2], zeolites have been widely used for environmental protection purposes: the decontamination of tap and wastewater [3], heavy metals and organic contaminants sorbents [4,5], slow release fertilizers [6], carriers of herbicides, fungicides, and pesticides [7], and/or soil conditioners, improving carbon sequestration [8,9], soil structure, and water storage [10,11]. Natural zeolites have a negatively charged surface, so they have been commonly applied to manage cationic nutrients concentrations in soil, preventing to some extent their slow and gradual leaching from the soil profile [12]. Nitrogen, a nutrient critical to plant growth, in well-aerated agricultural soils occurs mainly in the anionic form of nitrate (ammonium or amide nitrogen forms are easily oxidized), which is repelled by negatively charged soil colloids and is easily transported out of the rhizosphere [13]. To adsorb nitrate, zeolites are applied also; however, their surface is chemically modified with various positively charged species to enable anion adsorption [14,15]. Strong- or weak-base organic anion exchangers are commonly used for this purpose [16]; however, synthetic resins are often not suitable due to their potential hazard as another contaminant source, and those synthesized from natural products [17,18] are generally not stable under soil conditions. For more environmental-friendly applications, zeolites are modified with metal cations or metal oxides in simple, effective, and relatively inexpensive procedures [19,20]. The charge properties of modified zeolites depend both on the kind of the modifier and conditions of preparation [21]. Calcination at rather high temperatures is frequently applied [22,23]. Modified zeolites exhibit a high ability to adsorb soil organic matter [24] that may be important for carbon sequestration. Another advantage of zeolites is their ability to store large amounts of water, which is particularly important during events of water deficit that reduce crop production [25,26]. The use of zeolite in drought periods has a significant effect on yield and the physicomorphological characteristics of plants [27,28,29]. In this paper, different modifications of a zeolite (including washing and calcination) with iron, aluminum, and hydrogen ions, compounds of which are present in huge amounts in all natural soils, were investigated. Since most of the research relates to the surface area and microporosity, here the fractal dimension, adsorption energy, and mesopore parameters were studied additionally. Reports on the latter properties are hardly available in the literature; despite that, they may have an important effect on water storage and its availability for plants. The second aim was to estimate the electrokinetic (zeta) potential of modified zeolites in a wide range of pH. We could not find in the literature such a systematic approach to modified zeolites characteristics. In the present paper, we tried to fill both the above knowledge gaps by studying the effect of various modifications on the physicochemical properties of a clinoptilolite.

## 2. Materials and Methods

### 2.1. Preparation of Zeolite Samples

**Figure 1:**
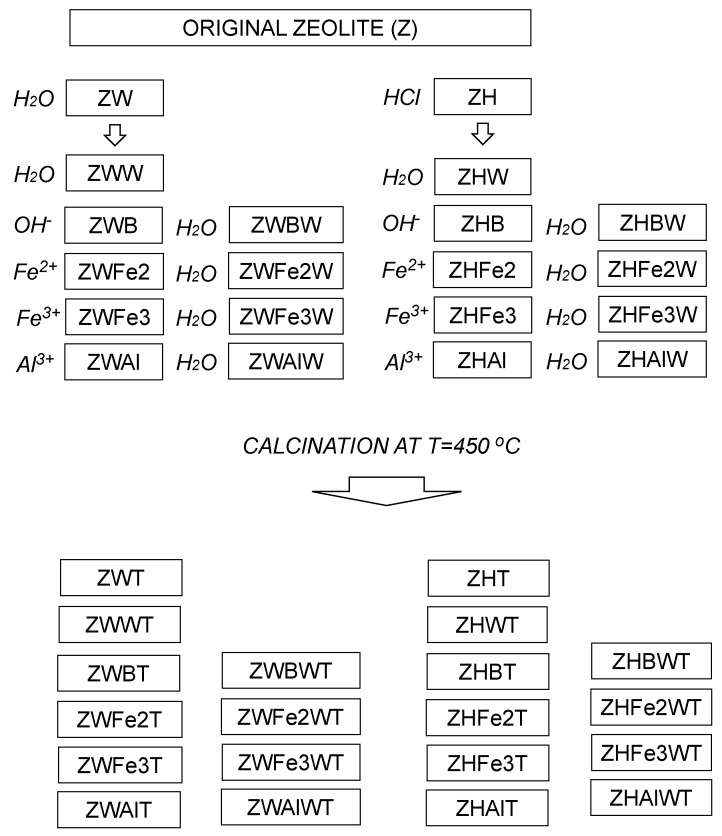
Scheme of the sample preparation. The treatments are written in italic. Abbreviation of the samples obtained after each treatment are closed within rectangles.

Smaller than 0.5-mm diameter fraction separated by sieving from ground Transcarpathian clinoptilolitic tuff localized in Sokyrnytsya, Ukraine [30] was modified according to a procedure described by Swiderska-Dabrowska et al. [22] with some modifications. First, the original zeolite (Z) was pre-treated with 5 bedvolumes of water (ZW) or 5% HCl (ZH) in a reciprocal shaker for 11 h. The treatment media were renewed three times during the first 6 hours (every 2h), and the solid phase was separated by filtration and dried at 105 °C. Parts of the obtained materials were washed thrice with 10 bedvolumes of distilled water (ZWW and ZHW), filtered and 105 °C dried. Next, both ZW and ZH were shaken for 4 h with 5 bedvolumes of 0.1 M solutions of FeSO_4_, FeCl_3_ or AlCl_3_, filtered, adjusted finally to pH 9.0 with 0.1 M NaOH, filtered again, and 105 °C dried (the respective procedures are abbreviated as Fe2, Fe3, and Al). Parts of the above materials prior to drying were washed thrice with 10 bedvolumes of water (abbreviation with additional letter W at the end). The treatment with NaOH (adjustment to pH = 9 with and/or without the final washing) was performed also for not metal cations—modified materials—ZW and ZH (the respective samples are abbreviated with letters B—or BW, if finally washed). Samples of all modified zeolites were additionally calcined at the temperature T = 450 °C (calcination is abbreviated with the letter T). The calcination temperature was chosen according to Swiderska-Dąbrowska [22,23], who obtained the best Fe-modified zeolite at 450 °C (point of zero charge at pH 7 and high values of zeta potential over a wide pH range), whereas 350 °C calcination resulted in lowest values of zeta potential. The scheme of the modification procedure is presented in Figure 1.

### 2.2. Measurements and Analysis of Water Vapor Adsorption Isotherms

Adsorption/desorption isotherms were estimated in triplicate for microsamples (~20 mg) of the studied materials using a DVS Intrinsic apparatus provided by Surface Measurement Systems Ltd, London, UK at 20 °C at the relative water vapor (p/p_0_) range of 0.06–0.97. The dry mass of the samples was estimated after overnight drying under the nitrogen atmosphere.

The adsorption data were used to calculate the specific surface area S (m^2^ g^−1^) using the linear form of the Aranovich [31] equation:

*x/a*(1 − *p*/*p*_0_)^1^^/2^ = 1/(*a*_m_*C*) + *x*/*a*_m_, (1)

where *x* is *p/p*_0_, *a* (kg kg^−1^) is the amount of adsorbed water at a given *x*, *a*_m_ (kg kg^−1^) is a monolayer capacity, and *C* is a constant.

The surface area was calculated as:

*S* = *a*_m_ω*L*/*M*, (2)

where *a*_m_ was estimated from the slope of Equation (1), *ω* is the area of a single water molecule (10.8 × 10^−19^ m^2^), *L* is the Avogadro number, and *M* is the molecular mass of water.

To find adsorption energy, it was assumed that the real adsorbing surface is a combination of energetically homogeneous patches of number *i* [32,33] having distinct energies *E*_i_ = (*E*_a,i_-*E*_c_), where *E*_a,i_ is the adsorption energy of *i*-th patch. Then, the total water vapor adsorption at a given pressure, *a*(*p*), was expressed as a sum of local adsorptions *a*_i_ on different patches:

(3) $$a(p)=\sum_{i=1}^{n}a_{i}(p,E_{i}),$$

where *n* is the total number of patches. Thus, the total adsorption isotherm, Θ(*p*), is a sum of adsorptions on all patches, Θ _i_(*p*,*E*_i_), weighted by their fractions, f(*E*_i_):

(4) $$\Theta (p)=a(p)/a_{m}=\sum_{i=1}^{n}a_{i}(p,E_{i})/a_{m,i}(a_{m,i}/a_{m})=\sum_{i=1}^{n}\Theta_{i}(p,E_{i})f(E_{i}),$$

where *a*_m,i_ is the monolayer capacity of the patch kind *i*, and values of f(*E*i) fulfill the normalization condition:

(5) $$\sum_{i=1}^{n}f(E_{i})=1.$$

To find f(*E*_i_), a condensation approximation [34,35] was applied, replacing the true local isotherm by a step function, arriving at the final formula:

f(*E*_i_) = [(1-*x*_i+1_)1/2 Θ(*E*_i+1_) - (1-*x*_i_)1/2 Θ(*E*_i_)]/(*E*_i+1_ - *E*_i_), (6)

where *E* = -RTln(*p*_0_/*p*).

Having calculated f(*E*_i_) values, the average water vapor adsorption energy of the whole adsorbent, *E*_av_, was calculated as:

(7) $$E_{\mathrm{av}}=\sum_{i=1}^{n}E_{i}\;f(E_{i}).$$

Energy values were expressed as positive scaled energies showing an excess of adsorption energy, *E*_a_, over the condensation energy of water, *E*_c_, in units of thermal energy, RT: *E* = (*E*_a_-*E*_c_)/RT (R is the universal gas constant and T is the temperature of the measurements). The scaled energies of the adsorbing patches ranging from 0.0 to 3.0 were considered. The scaled energy equal to 0 holds for adsorption energy equal to the condensation energy. The value of 3.0 was taken as the maximum adsorption energy, because in performed experiments, the minimum *p*/*p*_0_ was ~0.06, which corresponds to the adsorption energy of around 2.8. However, this value should be considered only as a first estimate of the maximum energy because of the lack of experimental data at lower relative pressures.

To estimate mesopore radii, *r*, the Kelvin equation [36] relating the radius to the relative vapor pressure during desorption was used:

*r* = 2M γ cosα/[ρRT ln(p_0_/p)], (8)

where M is the molecular mass of water, γ is the water surface tension, α is a water–solid contact angle (assumed here to be zero), and ρ is the density of water.

This is frequently assumed that below *p*/*p*_0_, around 0.35 surface adsorption processes dominate, and the condensation processes occur at higher relative pressures; therefore, 1 nm was taken as a minimum rationale mesopore radius that corresponds to *p*/*p*_0_ = 0.342. The maximum mesopore radius was taken as 30 nm; this corresponds to *p*/*p*_0_ = 0.965, which is close to the maximum relative pressure applied in adsorption/desorption experiments.

The volume of the condensed liquid in the mesopores at a given pressure, *v*(*p*/*p*_0_)(m^3^), can be treated as a sum of pore volumes, *v*_i_(*r*_i_), of the radii *r*_i_ ≤ *r*(*p*/*p*_0_):

(9) $$v\left(p/p_{0}\right)=\sum_{i=1}^{n}v_{i}\left(r_{i}\right).$$

Dividing the above equation by the total pore volume, *v*_t_, the scaled desorption isotherm, Φ(*p*/*p*_0_)= Φ [*r*(*p*/*p*_0_)], can be treated as a sum of fractions of particular pores, f(r_i_):

(10) $$\Phi \left(p/p_{0}\right)=v\left(p/p_{0}\right)/v_{t}=\sum_{i=1}^{n}v_{i}\left(r_{i}\right)/v_{t}=\sum_{i=1}^{n}f\left(r_{i}\right)=1$$

and the pore fraction in a given range of pore sizes can be calculated as:

f(*r*_i,av_) = Φ(*r*_i+1_) - Φ(*r*_i_), (11)

where *r*_i,av_ denotes the arithmetic mean of *r*_i+1_ and *r*_i_.

The average pore radii, *r*_av_, in the measuring range was calculated as:

(12) $$r_{\mathrm{av}}=\sum_{i=1}^{n}r_{i,\mathrm{av}}\;f(r_{i,\mathrm{av}})$$

Adsorption data were used to evaluate surface fractal dimensions, *D*, from a linear part of the dependence [37,38]:

ln(a) = C − (1/m)ln[-ln(*p*/*p*_0_)], (13)

where C is a constant, and the parameter m is related to the surface fractal dimension of the sample.

The magnitude of the parameter 1/m distinguishes two possible adsorption regimes: when 1/m <1/3, the adsorption occurs within van der Waals regime, and the surface fractal dimension is then D = 3(1 − 1/m). Alternatively, for 1/m >1/3, the adsorption is governed by the capillary condensation mechanism, and D = 3 − 1/m [32].

### 2.3. Determination of Electrokinetic (Zeta) Potential

Suspensions of the studied materials (1.5 g L^−1^) in distilled water filtered by a 0.02-µm membrane (Whatman, GE Healthcare UK Ltd., Little Chalfont, UK) were adjusted to pH values from 3 to 10 using 1 M HCl or 1 M NaOH. The electrophoretic mobility of dispersed particles was determined using a Zetasizer Nano ZS (Malvern Ltd., Malvern, UK) and the laser Doppler velocimetry method [39]. Measurements were performed at 20 ± 0.1 °C in six replicates. Zeta potentials were calculated using Henry’s equation [40]. From the dependence of zeta potential on pH, isoelectric points were estimated. 

## 3. Results and Discussion

**Figure 2:**
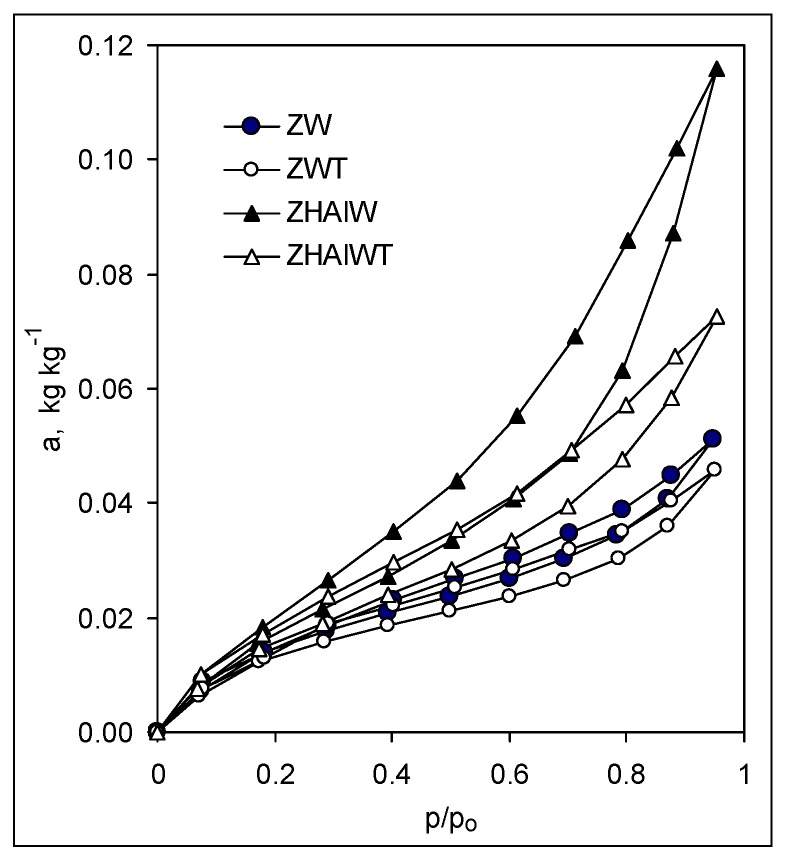
Exemplary adsorption–desorption isotherms. Abbreviation of the samples as in Figure 1.

The adsorption/desorption isotherms of the studied materials, presented exemplary in Figure 2, reflected in all cases a physical sorption process (second type in the IUPAC classification [41]) and exhibited in most cases well pronounced sorption hysteresis loops that terminate around p/p_0_ = 0.2.

Numerical values of the surface, mesopore, and electric charge parameters for the studied materials are presented in Table 1.

The specific surface area of the original zeolite was affected neither by distilled water washing nor by alkalization to pH = 9. Modification of the zeolite by protons and iron, and aluminum cations led to significant increase in surface area that most probably can be explained by the formation of surface-bound or individual precipitates of the metal hydroxides. This increase obeys the following order: Fe^3+^ (the average surface area for all modifications involving Fe^3+^ is 95 m^2^g^−1^) ˂ H^+^ (98 m^2^g^−1^ for only protons modifications) ˂ Al^3+^ (111 m^2^g^-1^ for all modifications involving Al^3+^) ˂ Fe^2+^ (117 m^2^g^−1^ for all modifications involving Fe^2+^). Note that the surface areas of zeolites modified with protons prior to Al^3+^ and Fe^2+^ cation treatments are larger than those materials that were not pre-treated with acid. The surface area of the proton-modified zeolite locates between Fe^3+^ and Al^3+^-modified materials. The acid treatment leads to Al dissolution from an aluminosilicate lattice [42] that supplies the reaction medium with an Al^3+^ modifier. Before adjustment of the medium to pH = 9, except for ion exchange processes, the precipitation of hydroxides is very likely to occur due to the neutral pH and some buffering properties of the studied zeolite. The order of the surface area increase coincides with the increasing solubility of the respective hydroxides. The lowest solubility has iron III hydroxide (the solubility product is around 10^−38^), then Al hydroxide (SP around 10^−33^), and the highest solubility has iron II hydroxide (SP around 10^−15^). It is possible that metal cations penetrate to some extent into inner parts of the crystal lattice of the zeolite and more soluble cations penetrate deeper; thus, more hydroxides of high surface area can precipitate, increasing the overall extent of an adsorbing surface. From the point of view of surface area, the modification of zeolites with protons and next by divalent iron cations seems most promising (ZHFe2 had the highest surface area). It is interesting whether the high surface area of Fe^2+^ modified zeolite remains not altered in time due to easy oxidation of Fe^2+^ to Fe^3+^. We would like to study this problem in the future.

The adsorption energies of the original, distilled water-washed as well as alkalized zeolites are higher than for the modified ones. Modifications of the zeolite decreased (on average) the adsorption energy in the following order: H^+^ (1.644) ≤ Al^3+^ (1.639) < Fe^2+^ (1.627) = Fe^3+^ (1.627). The energy of water sorption reflects the force of water binding. Zeolite added to the soil competes for water with soil constituents and plant roots. The distinction between a source and a recipient of water in such a system depends on the sorption energy of its particular components. Thus, an effect of modifiers on decreasing the sorption energy of zeolite may be of practical importance for the further application of the modified materials. In the above respect, iron-modified zeolites can be better than the other ones. It is also possible that changes to the sorption energy of polar water molecules reflect these for the other polar compounds, which along with surface areas may be important in catalytic processes involving modified zeolites and polar reagents.

The fractal dimension of the original, distilled water-washed as well as alkalized zeolites were higher than those for modified ones. Modification of the zeolite by iron and aluminum decreased on average the fractal dimension in the following order: H^+^ (2.54) < Fe^2+^ (2.48) < Fe^3+^ (2.45) < Al^3+^ (2.42). Since the fractal dimension can vary between 3 (rough and complicated surface) and 2 (flat, two-dimensional surface), it may indicate that the precipitated iron and aluminum hydroxides have smoother surfaces than the original zeolite. Smoother surfaces have usually lower adsorption energies, as it occurred for the studied adsorbents, as well. That the effect of protons treatment on both of the above parameters is smallest may indicate that the original surface of the zeolite is not much affected by a short time contact with protons and/or that eventual precipitation of lattice-originated aluminum hydroxide is not high.

The volume of the mesopores of the original zeolite was affected neither by distilled water washing nor by alkalization to pH = 9. Modification of the zeolite by protons and iron, and aluminum cations led to significant increase in mesopore volume, in the following order: H^+^ (44 mm^3^g^−1^) ˂ Fe^3+^ (51 mm^3^g^−1^) ˂ Fe^2+^ (56 mm^3^g^−1^) ˂ Al^3+^ (82 mm^3^g^−1^). Proton-treated zeolite has fewer mesopores than metal cations-modified materials, indicating that the input of the mesoporosity of the precipitated hydroxides is larger than that of additional mesopores produced after lattice dealumination. We expected that for metal cations-treated materials, the mesopore volume will increase with an increase in surface area (adsorbents of a larger surface area have generally higher mesopore volumes). No parallel changes of the above parameters may be related to the different crystallinity and microstructure of the respective hydroxides and differences in their location on the zeolite surface. Higher mesopore volumes of the modified zeolites can make them more efficient water storage soil conditioners than the natural mineral. 

The mesopore radius of the original zeolite was not affected by distilled water washing. The positive effect of alkalization to pH = 9 on a mesopore radius (ZWB material) disappeared practically after final washing (ZWBW). Modification of the zeolite by aluminum cations led to an increase in the average mesopore radius (8.59 nm). A similar effect was observed for proton modifications (8.44 nm). However, the modification with iron cations led to a decrease of mesopore radius (7.77 nm for Fe3 and 7.69 nm for Fe2). Differences in mesopore radii behavior may be due to structural differences of the respective hydroxides, similarly as for mesopore volumes. Linking surface areas and mesopore characteristics to the structural properties of hydroxides and hydroxides–zeolite associations seems to be a great scientific challenge. 

Zeolites have frequently been reported to assure a permanent water reservoir, holding water more than half of their weight due to the high porosity of crystalline structures. Water molecules in the pores could easily be evaporated or reabsorbed without damage to such structures. In prolonged moisture dry periods, zeolites help plants withstand dry spell; they also promote a rapid rewetting and improve the lateral spread of water into the root zone during irrigation. This results in saving water that is needed for irrigation. The amendment of sand with zeolite increases the available water to the plants by 50% [43]. However, because inner pores (channels) within a zeolite lattice have typical diameters of 0.5 to 0.7 nm, which is only slightly larger than the diameter of a water molecule, water present in the micropores cannot be used by plants at any conditions, and such high plant water supply properties seems to be a misinterpretation of the total zeolite porosity in terms of plant available water. We think that mesopores are much more important in this respect. The increase of mesopore volume accompanied by an increase in mesopore radii suggests that modified zeolites provide better water availability for plants than the original zeolitic tuffs. In the above respect, aluminum-modified zeolites are most promising.

**Figure 3:**
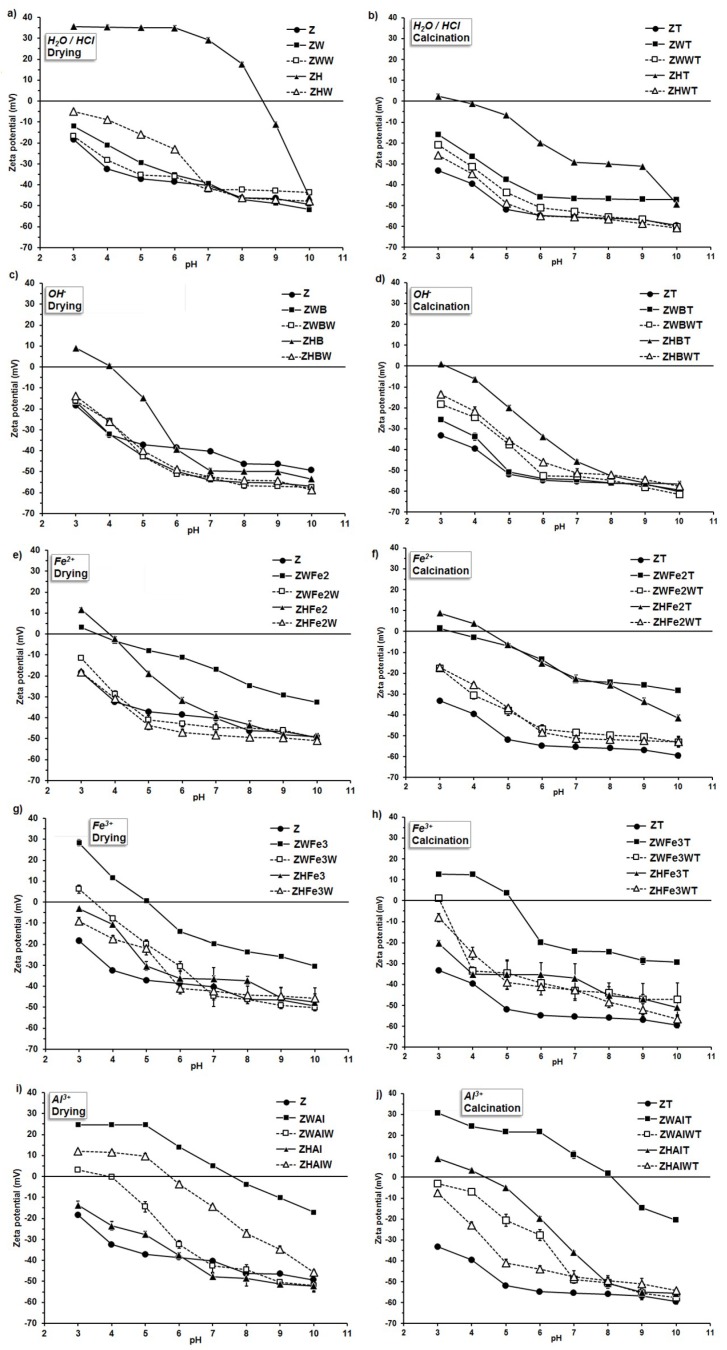
Zeta potential vs. pH dependence for the zeolie mofified by (**a**) the pretreatment with H_2_O or HCl; (**b**) the pretreatment with H_2_O or HCl and calcination; (**c**) the alkalization; (**d**) the alkalization and calcination; (**e**) the Fe^2+^ action; (**f**) the Fe^2+^ action and calcination; (**g**) the Fe^3+^ action; (**h**) the Fe^3+^ action and calcination; (**i**) the the Al^3+^ action; (**j**) the Al^3+^ action and calcination. The abbreviations of the sample names are in accordance to Figure 1.

The zeta potential of the studied materials varied with the pH of the external environment, which is illustrated in Figure 3. 

The original zeolite revealed negative values of zeta potential in the whole experimental pH window (i.e., 3–10). The net surface charge of this mineral results from its negatively charged crystal structure, and it is neutralized by exchangeable bonded cations of Na, K, Ca, Mg, and Fe [44]. Linear extrapolation of potential versus pH data gave the isoelectric point (IEP) of natural zeolite at a pH of 0.76–0.88. After FeSO_4_ treatment, the IEP of zeolite increased to 1.13–4.38. The use of FeCl_3_ resulted in an IEP range of 1.63–4.97, whereas AlCl_3_ gave the values of 1.35–7.91 (see Table 1). Similarly, Nguyen et al. [45] reported that the IEP of the iron-coated zeolite dispersed in 10^-3^ M NaNO_3_ was 5.6, whereas it was 2.2 for natural material. Guaya et al. [20] found that zeolite modified by aluminum had the point of zero charge (PZC) at pH 4.5, and exhibited good ability for the simultaneous sorption of ammonium and phosphate from solution. In addition, Chen et al. [46] observed positive zeta potentials due to an increase of the Al/Si ratio in zeolite. The IEP of metal cations-modified zeolites usually locate at higher pHs than these modified by organic compounds. Mahmoodi and Saffar-Dastgerdi [21] modified natural zeolite by (3-aminopropyl) triethoxy silane, finding that the modified materials had positive zeta potentials at very low pHs (below 2). However, Arora et al. [13] observed nitrate adsorption on chitosan-modified zeolite at intermediate pH values. It can be seen (Figure 3) that proton-modified zeolite (ZH sample) had the highest isoelectric point (pH = 8.6). This is difficult to explain, because acidification should lead to more negative values of zeta potential due to the Si–O bond strength modification, and a decrease in Al/Si ratio due to lattice dealumination [44]. Probably, the dissolved Al ions were not able to diffuse outside the mineral lattice under the applied experimental conditions. They may adsorb and precipitate inside the lattice, forming positively charged internal coatings. Small protons can penetrate easier and deeper into the lattice spaces than larger aluminum cations during Al modification, and therefore, the effect of protons may possibly be higher. Positive values of zeta potential may be also connected with strong H^+^ adsorption, which is enlarged due to the presence of defects in zeolite crystal after Al removing, as postulated by Wang and Nguyen [47].

Some modified zeolites, which were obtained in the present research, exhibited isoelectric points at rather high pH values (ZWAl at 7.5; ZWAlW and ZHAlW at 4.0; ZWFe3 at 5.4; and ZHFe3 at 4.5). Such materials may be particularly useful for nitrates management in soil, not losing at the same time their cationic nutrients retention properties. As proved by Northcott et al. [48], besides anion exchange, the modified zeolites can keep the ability to adsorb inorganic cations as well, because the modifiers are relatively large molecules, and remain on the external surface of the zeolite crystals and do not enter zeolite channels.

**Figure 4:**
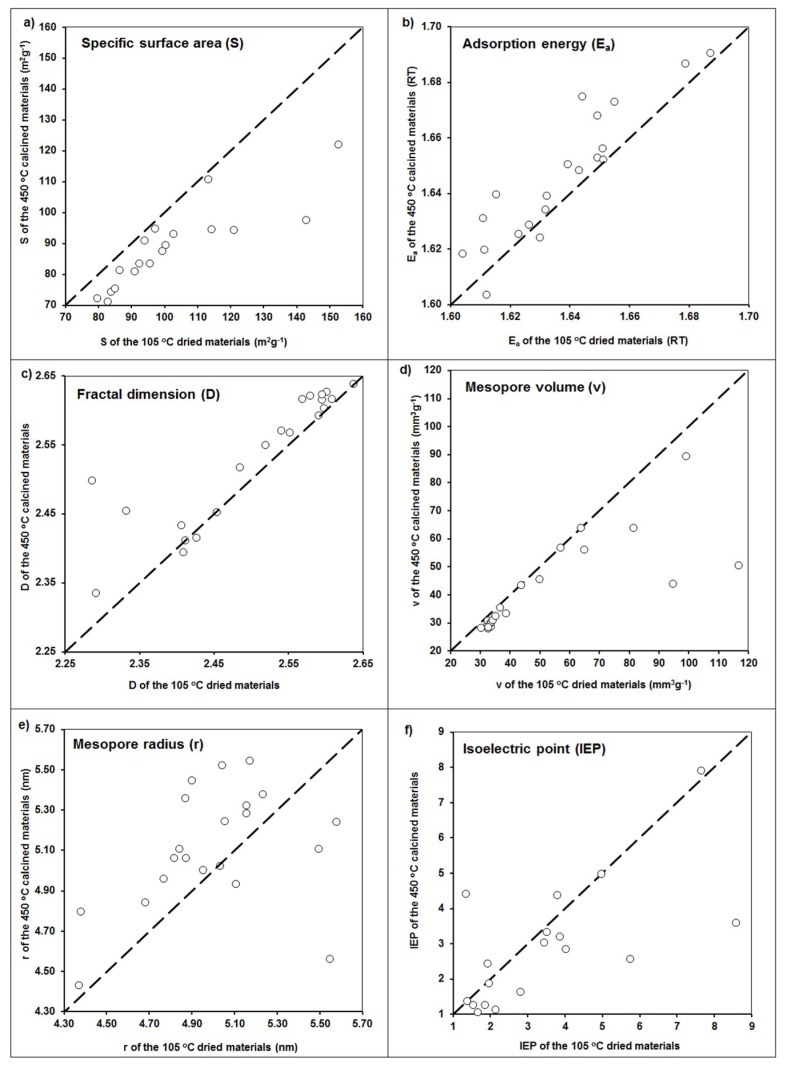
Changes in (**a**) specific surface area, (**b**) adsorption energy, (**c**) fractal dimension, (**d**) mesopore volume, (**e**) mesopore radius, and (**f**) isoelectric point values after calcination of the studied materials. Dashed lines are 1:1 dependencies.

In general, some trends are valid for all the modifications that were observed. The samples washed with distilled water after the application of the modifiers, as compared to unwashed samples, had smaller surface areas and mesopore volumes, higher mesopore radii and fractal dimensions, and more negative zeta potential values. Exactly the same trends were observed after materials calcination, which are exemplary presented in Figure 4.

That a final washing of the modified materials leads in general to a decrease in the surface area, amount of mesopores, and shift in surface potential toward more negative values seems obvious, because a part of the modifier was washed out. As it can be seen (Figure 4), calcination resulted in similar effects: a decrease of specific surface area, mesopore volume, and value of isoelectric point, as well as an increase of both the adsorption energy and mesopore radius. This may be due to the dehydration of surface hydroxyls during calcination. However, an increase in the binding strength between the hydroxide modifier and the zeolite surface during calcination may increase the stability and mechanical resistance of the modified zeolites [23], which is an advantageous feature for the modified zeolites’ applications. 

## 4. Conclusions

In this paper we applied water vapor adsorption and electrokinetic measurements to study the impact of iron, aluminum, hydrogen, and hydroxyl ions modifiers, as well as post-modification washing and calcination on the surface and charge properties of a zeolite. Practically, all the applied modifiers increased the zeolite surface area, mesopore volume, and radius, and decreased the water-binding forces. Unwashed materials modified with Al and H cations had isoelectric points at rather high pH values (above 7). Calcination of the modified materials shifted their zeta potentials toward more negative values, diminished both surface areas and mesopore volumes, and increased mesopore radii. We see a need to draw more mechanistic explanation of the observed phenomena using a larger set of instrumental methods (e.g. XRD, SEM-EDS, FTIR, and NMR) supported by chemical analysis. Modified zeolites of positive surface charge can be applied for the anionic nutrients management in soils, sorbents of anionic contaminants in water purification plants, and binders of humic substances in soils (carbon sequestrators). Large surface areas and mesopore volumes of the modified zeolites give them more perspectives for applications as soil conditioners improving water retention. 

## Figures and Tables

**Table 1:** Specific surface area (S), fractal dimension (D), adsorption energy (Ea), mesopore volume (v), average mesopore radius (r), and isoelectric point (IEP) of the modified zeolites.

Material	S (m^2^g^−1^)	D	Ea	v (mm^3^g^−1^)	r (nm)	IEP
Z	83.9 ± 2.1	2.55 ± 0.07	1.626 ± 0.04	33 ± 1.0	8.02 ± 0.5	0.88 ± 0.07
ZW	79.1 ± 1.7	2.57 ± 0.05	1.655 ± 0.03	33 ± 0.9	7.98 ± 0.5	1.38 ± 0.01
ZWW	79.3 ± 2.0	2.60 ± 0.03	1.679 ± 0.02	34 ± 1.0	8.03 ± 0.7	1.11 ± 0.02
ZH	95.6 ± 1.3	2.48 ± 0.06	1.639 ± 0.03	50 ± 0.4	8.34 ± 0.4	8.59 ± 0.08
ZHW	99.5 ± 2.5	2.59 ± 0.05	1.649 ± 0.03	39 ± 1.2	8.55 ± 0.6	2.33 ± 0.05
ZWB	82.9 ± 1.3	2.58 ± 0.06	1.644 ± 0.03	32 ± 0.8	8.35 ± 0.6	1.66 ± 0.03
ZWBW	81.3 ± 1.7	2.59 ± 0.07	1.649 ± 0.04	30 ± 0.6	8.07 ± 0.2	1.87 ± 0.06
ZHB	92.4 ± 2.2	2.52 ± 0.10	1.611 ± 0.06	37 ± 1.0	7.93 ± 0.4	3.86 ± 0.11
ZHBW	91.1 ± 1.7	2.61 ± 0.07	1.651 ± 0.04	34 ± 0.5	8.53 ± 0.6	1.97 ± 0.02
ZWFe2	113.3 ± 2.9	2.45 ± 0.02	1.621 ± 0.01	57 ± 1.1	7.82 ± 0.3	3.52 ± 0.07
ZWFe2W	86.5 ± 1.8	2.60 ± 0.02	1.651 ± 0.01	34 ± 0.9	8.18 ± 0.6	2.15 ± 0.02
ZHFe2	152.7 ± 6.3	2.29 ± 0.07	1.623 ± 0.03	99 ± 3.7	7.05 ± 0.5	3.80 ± 0.04
ZHFe2W	114.3 ± 2.5	2.59 ± 0.10	1.611 ± 0.06	35 ± 1.1	7.72 ± 0.7	1.55 ± 0.01
ZWFe3	102.7 ± 1.8	2.43 ± 0.01	1.630 ± 0.01	64 ± 1.0	8.30 ± 0.6	4.97 ± 0.10
ZWFe3W	85.1 ± 2.7	2.54 ± 0.05	1.632 ± 0.02	33 ± 1.6	7.94 ± 0.3	3.45 ± 0.03
ZHFe3	93.9 ± 4.4	2.41 ± 0.01	1.612 ± 0.01	44 ± 2.8	7.18 ± 0.5	2.81 ± 0.14
ZHFe3W	97.3 ± 4.6	2.41 ± 0.05	1.632 ± 0.03	65 ± 3.0	7.67 ± 0.4	1.94 ± 0.06
ZWAl	100.4 ± 6.9	2.41 ± 0.01	1.648 ± 0.01	82 ± 5.3	8.41 ± 0.6	7.66 ± 0.08
ZWAlW	79.7 ± 2.1	2.64 ± 0.02	1.687 ± 0.01	33 ± 1,2	9.30 ± 0.6	4.02 ± 0.04
ZHAl	143.0 ± 11.6	2.29 ± 0.01	1.615 ± 0.01	117 ± 9.6	7.96 ± 0.8	1.35 ± 0.03
ZHAlW	121.0 ± 1.9	2.33 ± 0.01	1.604 ± 0.01	95 ± 1.3	8.70 ± 0.3	5.76 ± 0.06
